# Designing a zinc–calcium selenite nanocomposite: unmasking structural, morphological, optical, magnetic and electrochemical characteristics with enhanced supercapacitor performance

**DOI:** 10.1039/d6ra05141c

**Published:** 2026-07-17

**Authors:** S. Ariponnammal, R. Harshinee, S. Shanmugha Soundare, M. Mithren

**Affiliations:** a Department of Physics, Gandhigram Rural Institute, Deemed to Be University Gandhigram-624302 Dindigul District Tamilnadu India ariponnammal@gmail.com; b Centre for Nanoscience and Technology, Anna University Chennai-600025 Tamilnadu India soundaresivaumar@gmail.com

## Abstract

The hydrothermal method is used to create a zinc–calcium selenite (ZnSeO_3_/CaSeO_3_) nanocomposite. It displays nanorods that resemble capsules. The refractive index, energy gap, particle size, and Urbach energy is 1.972, 5.191 eV, 109.4 nm, and 0.621 eV, respectively. The sample shows interesting UV filtering and optoelectronic properties. It is a material with potential for display technologies because spectroscopic research has revealed native and surface point defects that promote radiative electron–hole recombination. The distinctive bands Zn–O, Se–O, and Zn–Se are revealed by infrared spectroscopy. At 300 K, the sample shows modest ferromagnetic behaviour. A thermal study demonstrates the material's behavior, thermal stability, and decomposition at different temperatures. XPS guarantees the orbital state of an element with spin–orbit coupling (*j*) and validates the chemical state. With a potential window of 1 V and a specific capacitance of 862.81 F g^−1^, electrochemical analysis shows pseudo-capacitive activity.

## Abstract 2

The hydrothermal method is used to create a zinc–calcium selenite (ZnSeO_3_/CaSeO_3_) nanocomposite.

## Graphical abstract

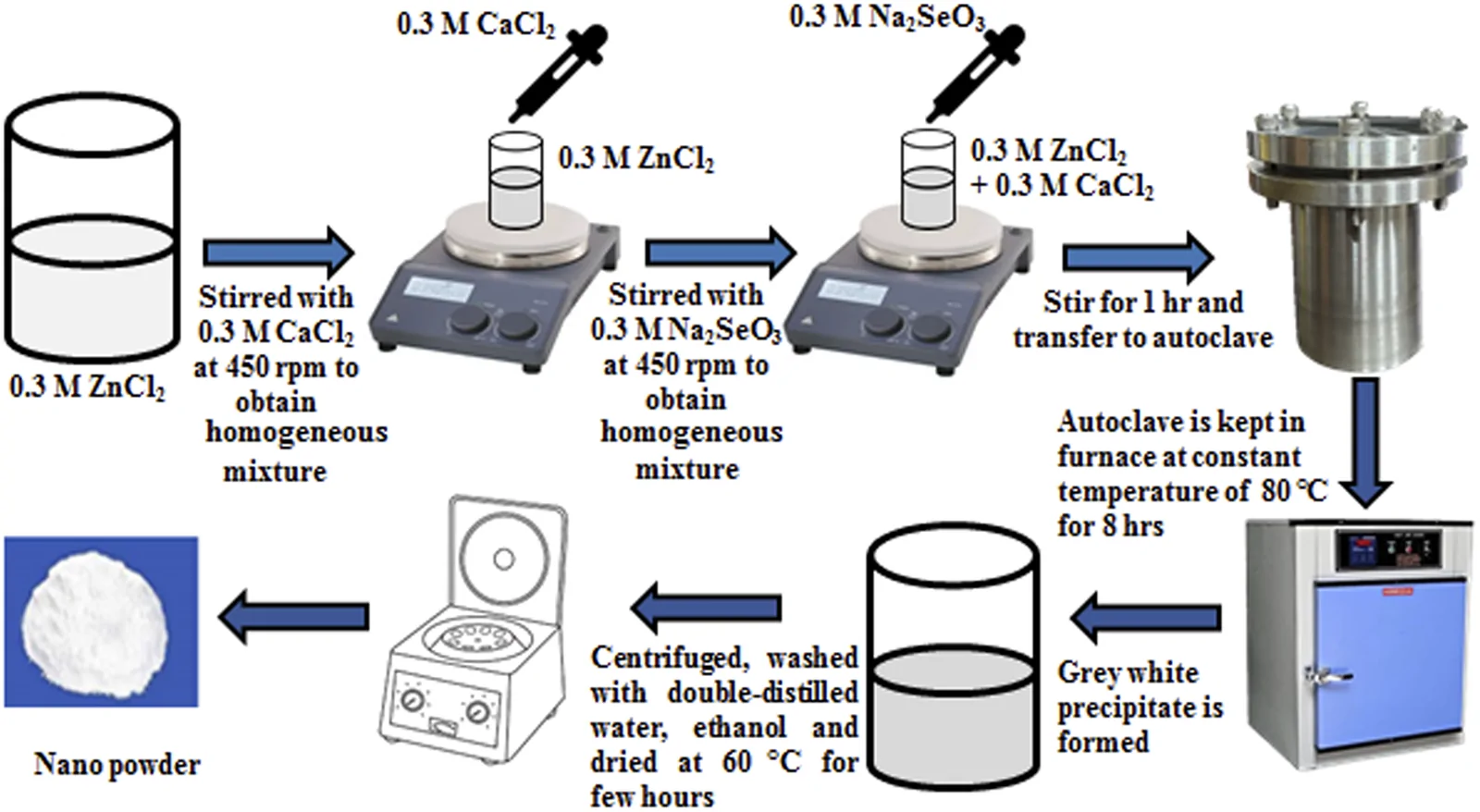

## Introduction

Conventional and non-conventional energy sources are two primary energy sources. The development of efficient energy storage technologies is a pressing requirement nowadays. Energy must be enormous for uninterrupted supply.^1^ Fossil fuel energy causes adverse environmental effects, such as global warming and the climate crisis. Renewable energy sources, such as solar, wind, and geothermal, have been introduced to address the environmental crisis.^2–4^ In the clean energy portfolio, electrochemical energy is an essential one because of the high specific capacitance, extended life cycle, comparatively minimal maintenance, lack of memory effect, high power density, and safety of batteries, supercapacitors, and fuel cells. They provide an efficient method for supplying energy in remote areas without public grids. In addition to protecting batteries from rapid charge–discharge cycles and improving energy efficiency, electric and hybrid vehicles offer the high power density needed for short-term acceleration and energy retrieval during braking.^5^ The electrical performance of electrode materials is currently improved using supercapacitor technology. Supercapacitors are utilized in drive systems that take power from a voltage source to provide the power requirements for braking, energy recovery, and starting. As a result, it is an effective approach for many energy storage applications, such as backup power sources to protect against shortages. Many factors influence the performance of a supercapacitor, including the electrode's potential window, the electrode material's electrochemical performance, and the electrolyte. It has been reported that a straightforward method creates NPS-HPC (hierarchical porous carbon doped with N, P, and S) using a combination of doping from rice husk and potassium hydrogen carbonate activation. It has 1976.8 m^2^ g^−1^ exceptionally large surface area. Its thin sheet structures and appropriate content of heteroatoms provide active sites, facilitate rapid electron transport, and increase the redox interaction. The zinc ion hybrid capacitor (ZIC) based on NPS-HPC achieves excellent energy and power density due to structural advantages. Additionally, ZIC based on NPS-HPC exhibits a long cycle life of up to 23 000 cycles.^6^ Further, cooperative activation of KHCO_3_ and Na_2_S_2_O_3_ synthesizes N, S co-doped porous carbons with well-developed pores and an enormous surface area from natural biomass. These carbons exhibit high specific capacity and energy density in an aqueous two-electrode system.^7^

Electrode material engineering improves the electrochemical stability window and enhances electrochemical kinetics by providing strong interfacial interactions and abundant active sites. The hierarchical MnCO_3_/NiO heterojunction obtained by a one-pot, microwave method is a highly active electrode for aqueous supercapacitors. Strong interfacial contacts are used to optimize surface reaction and charge transfer processes, speed up redox kinetics, and enhance electron transport. Advanced MnCO_3_-based hetero junctions produced using a variety of techniques have proven to be widely applicable in the conversion and storage of energy. For instance, MgCO_3_@MnCO_3_@Mn_3_N_2_ heterojunctions have been developed for zinc-ion batteries with outstanding performance, while CuS/MnCO_3_ has demonstrated efficient electrocatalytic activity for water splitting. Similarly, Cu–MnCO_3_–Mn_3_O_4_/C heterostructures have shown greater catalytic activity for tetracycline degradation. In addition, NiFe-LDH/MnCO_3_/MXene, carbon-coated MnCO_3_@MnO_2_, MnCO_3_/Mn_3_O_4,_ and MnCO_3_@NiO nano-heterostructures have been reported to exhibit capacitances of 2079.6 F g^−1^, 267 F g^−1^, 191 F g^−1^, and 624 mAh g^−1^ at 1 A g^−1^, respectively.^8^ Binder-free (NiO)_0.75_ (MnO)_0.25_ porous nanosheet grown on nickel foam combines increased pseudocapacitance and a wider voltage window.^9^ Research on building a solid solution of nickel–manganese oxide with microwave assistance and simultaneously improving the voltage window and pseudocapacitance for an aqueous supercapacitor is going on. This research focuses on designing an advanced electrode material by combining cobalt oxide Co_3_O_4_ and lanthanum fluoride LaF_3_. The architecture integrates three distinct dimensionalities, 1D, 2D, and 0D, to create a heterojunction. This unique structure synergistically improves energy storage performance. A 1D–2D/0D Co_3_O_4_/LaF_3_ heterojunction is built to improve aqueous supercapacitors' capacity, rate capabilities, and voltage window all at once.^10^ MnO_2_/CNT heterostructures improve the performance of asymmetric supercapacitors.^11,12^ Mn_0.5_Co_2.5_O_4_ tailors structural and electrochemical properties for energy and catalysis applications.^13^

Therefore, research is ongoing to create sophisticated materials for supercapacitor electrodes with suitable structural designs that allow for the best possible ionic diffusion and electron transport. Therefore, the current effort focuses on charge storage mechanisms in materials of electrode metal oxychalcogenides, metal chalcogenides, and perovskite materials to develop new electrodes to improve the supercapacitor performance.^14^ In high-power-density applications, supercapacitors (SCs) offer a number of benefits, but their extended lifespan necessitates multiple cycles of charging and discharging. SCs are not appropriate for alternating currents (AC) and are usually used for shorter periods of time, from low power to high power. Consumer electronics, microgrids power supply, stabilization of voltage, renewable energy storage, harvesting of energy, street lights, therapeutic applications, military, powering portable devices like notebook computers, cell phones, and digital cameras, and energy recovery are just a few of the uses of supercapacitors.^1,15^

The synthesis of nanoparticles remains a significant challenge in the development of multi-functional materials, as precise control over particle size and morphology is essential for achieving unique properties and enhanced performance. The synthesis method in this present work is the hydrothermal method, which is very effective in controlling particle size.^16^ Zinc selenite has unique physicochemical properties and potential multifunctional agent. It is an essential material for short-wavelength applications. Its high luminescence, low coefficient of absorption, wide band gap, transmittance range, and superior transparency to infrared radiation make it suitable for various applications, including optical instruments, photovoltaics, laser screens, blue laser diodes, and high-speed optical devices.^17^ Several attempts have been made to improve the efficacy of the materials by substitutional doping, changing the synthesis technique, and annealing at different temperatures and times.^18^ Studies based on zinc and calcium systems have been scarce because calcium has a greater ionic size than zinc, whereas calcium is particularly useful for lattice stabilization and enhances the ionic character of bonding in ZnO systems. Conversely, investigations based on the Zn–Ca system are being used, and therefore, in this study, the Zn–Ca system is synthesized and characterized in detail.

The main aim of the present study is to systematically investigate the characteristics of ZnSeO_3_/CaSeO_3_ synthesised *via* the hydrothermal method. These are expected to be used not only for selenium-based perovskites but also for a broader class of double perovskite oxides. All of these studies on ZnSeO_3_/CaSeO_3_ show that it is a versatile material that emphasizes a composite structural effect over a single characteristic.

This study's novelty provides a comprehensive correlation between structural alterations of selenium-based double perovskite oxides optical, magnetic, and electrochemical responses in ZnSeO_3_/CaSeO_3_, leading to the customization of band gap behaviour, chemistry of defects, and strain in the lattice.

This study increases our fundamental understanding of technological advancement. These lead to perovskite-based capacitors with better energy storage densities, the enhancement of multifunctional materials, and the improvement of absorption qualities, where characteristics can be simultaneously tuned.

## Methods and materials

**Fig. 1:**
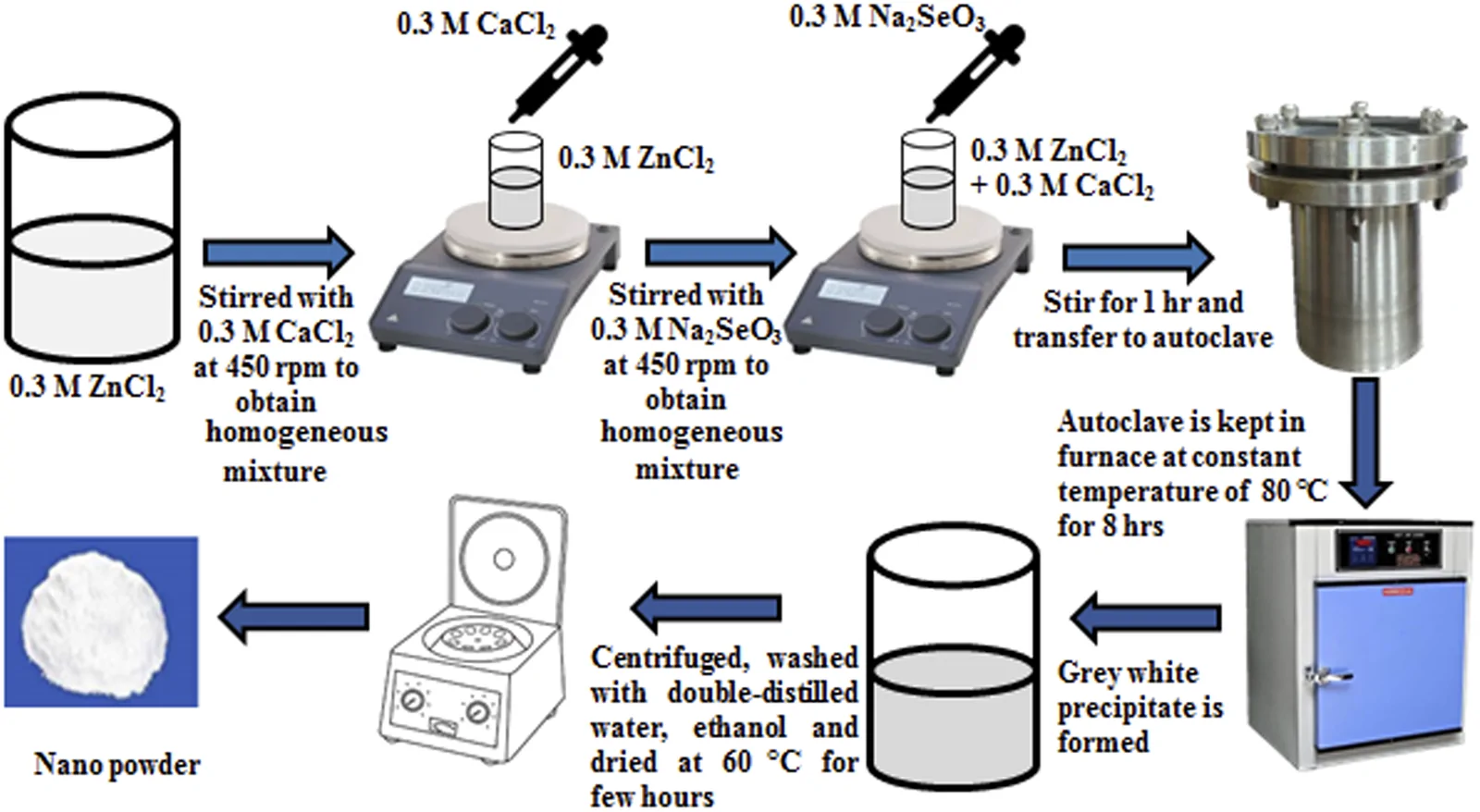
Synthesis of ZnSeO_3_/CaSeO_3_ nanocomposite.

The hydrothermal method (Fig. 1) is used to create the ZnSeO_3_/CaSeO_3_ nanocomposite. Analytical grade Alfa Aesar chemicals of ZnCl_2_, CaCl_2_, and Na_2_SeO_3_ are dissolved in 20 : 20 : 20 mL of double-distilled water individually to obtain 0.3 M each of ZnCl_2_, CaCl_2_, and Na_2_SeO_3_. Freshly made aqueous 0.3 M ZnCl_2_ solution was uniformly mixed with the solution 0.3 M CaCl_2,_ drop by drop, under continuous stirring at 450 rpm, to form a mixture of ZnCl_2_ and CaCl_2_. Then, 0.3 M Na_2_SeO_3_ is mixed with ZnCl_2_ + CaCl_2_ solution drop by drop, while being continuously swirled at 450 rpm. Then, the solution is kept in an autoclave at 80 °C for 8 h. After cooling, a grey-white precipitate is obtained. Then the precipitate is cleaned several times with deionized water and ethanol by centrifugation. The precipitate was kept in desiccant after being dried for a few hours at 60 °C.

Energy dispersive X-ray Analysis (EDAX) is depicted with the OXFORD INCAPENTAx3 model. The Panalytical X'Pert Pro records X-ray powder diffraction (XRD) with Cu Kα radiation, whereas the Carel ZEISS EVO-18 model reveals Scanning Electron Microscope (SEM). The UV-Vis spectrum is obtained using the PerkinElmer Lambda 35 spectrophotometer, while the Fourier Transform Infra Red (FTIR) spectrum is recorded with the PerkinElmer BX model, and magnetic measurements are performed with the Lakeshore VSM 7410 model. The NETZSCH NJA-STA2500 Regulus model with Proteus software is used for thermal analysis at temperatures between 30° and 1000° Celsius; the Thermo Fisher Scientific device uses Al Kα radiation (1486.6 eV) to record XPS spectra, and the Bio-Logic VSP potentiostat model is used for electrochemical investigations.

The electrochemical behavior of ZnSeO_3_/CaSeO_3_ was investigated in an electrochemical workstation using three electrode terminals in a 3 M KOH solution. The three-electrode configuration includes a working electrode made of nickel foam covered with a sample, an Ag/AgCl reference electrode, and a platinum wire counter electrode. Using a mortar and pestle, a solid mixture of 85 : 10 : 5 ZnSeO_3_/CaSeO_3_, carbon black, and PVDF was combined with a few drops of NMP solution (*N*-methyl pyrrolidone) to create the working electrode. A 1 × 1 cm^2^ area of the slurry-like substance was then placed over the pretreatment Ni foam (5% HCl/acetone/distilled water). After that, it was dried at 80 °C to generate the functional electrode. The electrode has a mass loading of 3 mg.

## Results and discussion

### Structure and surface morphology investigations

**Fig. 2:**
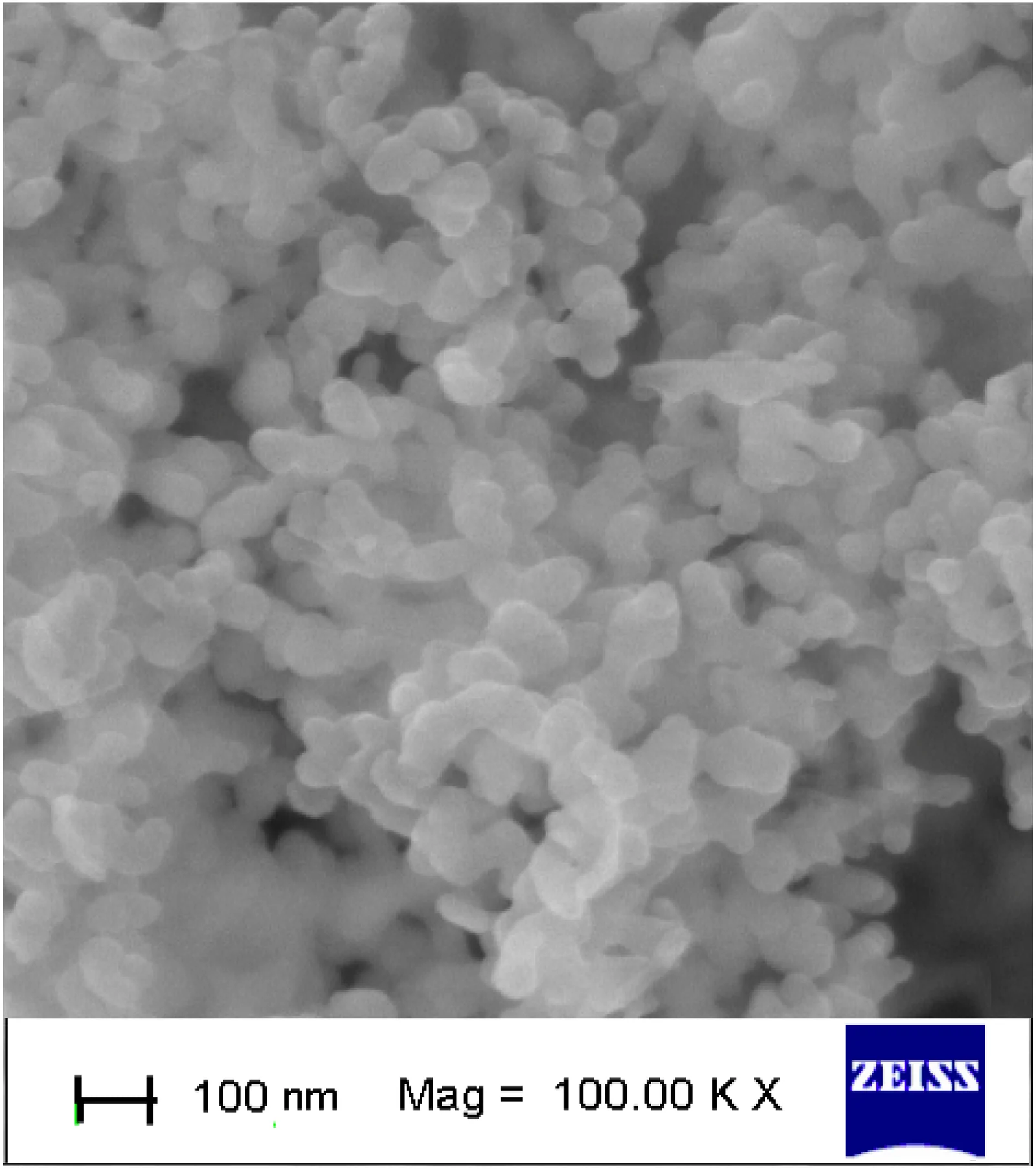
SEM image ZnSeO_3_/CaSeO_3_ nanocomposite.

The SEM image of ZnSeO_3_/CaSeO_3_ at 100 KX magnification (Fig. 2) clearly illustrates capsule-like nanorods. The observed capsule-like morphology represents a distinct transformation from spherical clusters, thereby influencing growth direction and surface energy. This change is not incidental but is closely linked to the surface activity during synthesis. It modifies the surface energy distribution and growth kinetics, selectively promoting elongation that leads to the formation of rod- or capsule-shaped particles.

These structures offer an enhanced surface area and may provide more continuous pathways for electron transport, better charge mobility, and reduced recombination, contributing to improved electrochemical performance.^19^ A particle size analyser measured the particle size at 109.4 nm.

**Fig. 3:**
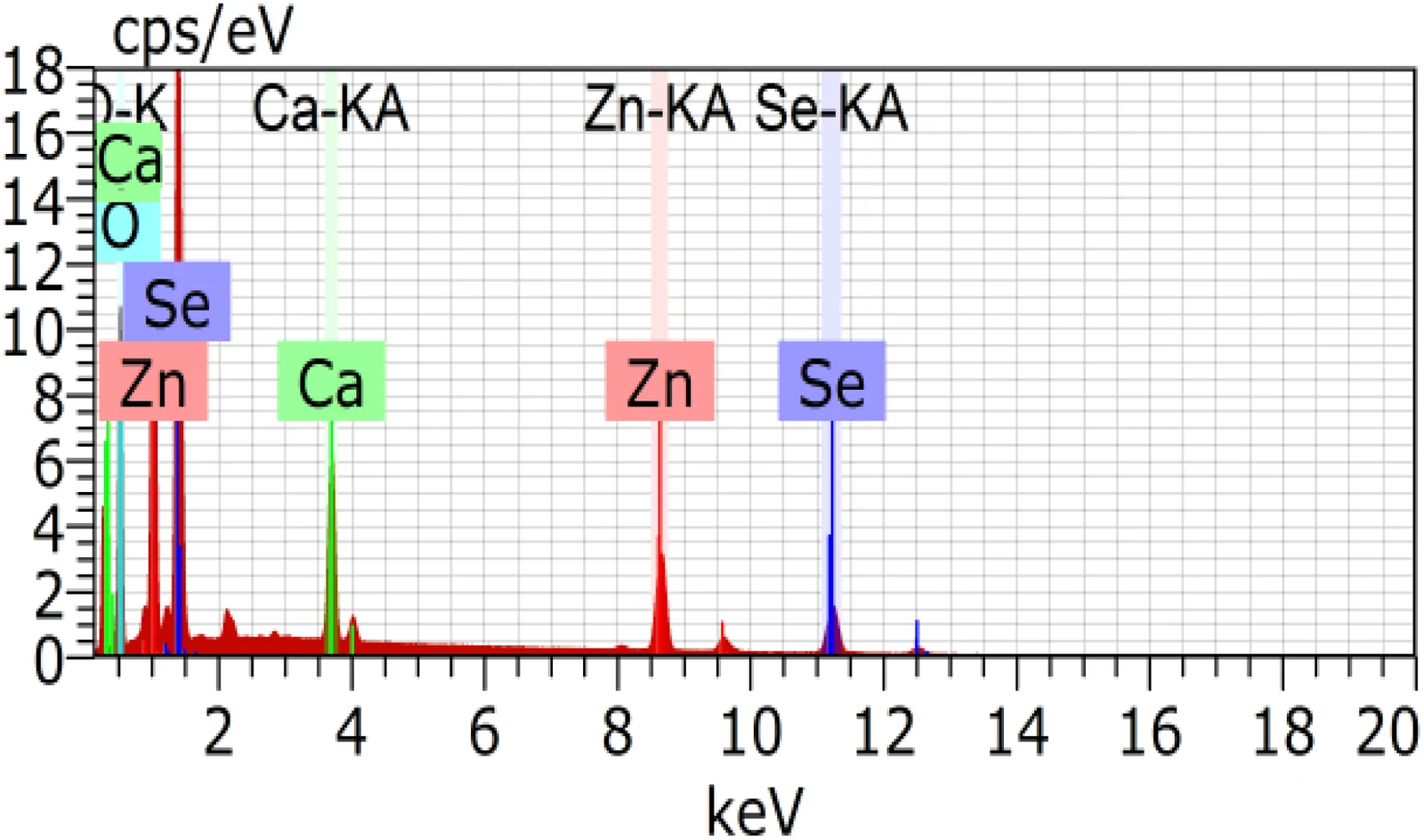
EDAX of ZnSeO_3_/CaSeO_3_ nanocomposite.

**Fig. 4:**
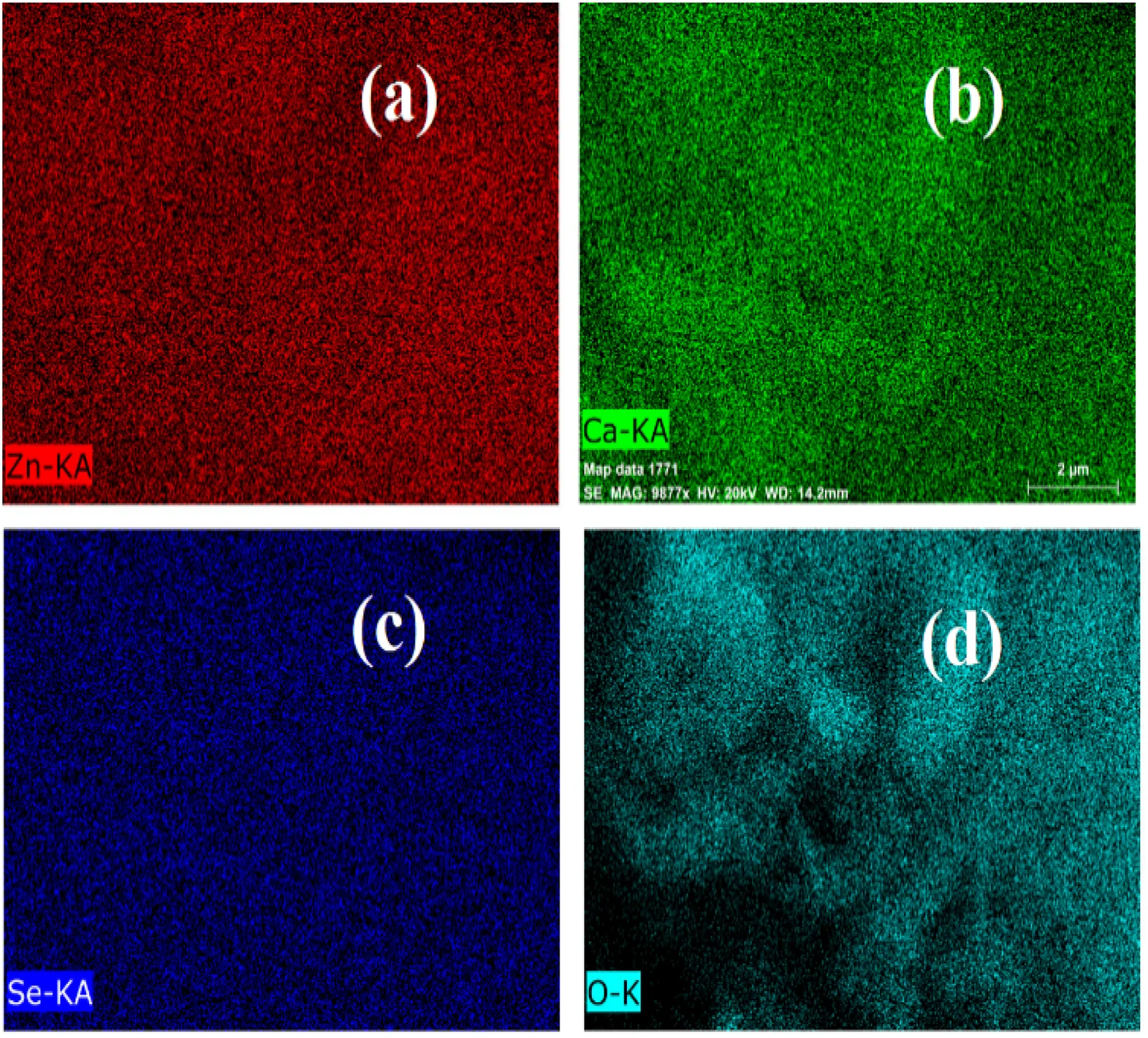
EDS image of (a) Zn, (b) Ca, (c) Se,and (d) O of ZnSeO_3_/CaSeO_3_ nanocomposite.

The EDAX of ZnSeO_3_/CaSeO_3_ is shown in Fig. 3. The weight percentages for zinc, calcium, selenium, and oxygen are 18.19%, 11.15%, 43.95%, and 26.71%, respectively, and match up with the computed values of 18.03%, 11.20%, 44.35%, and 26.42%, confirming the sample composition. Fig. 4a–d depicts the EDS of ZnSeO_3_/CaSeO_3_. Zinc, calcium, selenium, and oxygen elements make up the majority of the nanocomposite, as per mapping (Fig. 4a–d). This demonstrates the perfect formation and complete reaction of the sample.

### Structural study

**Fig. 5:**
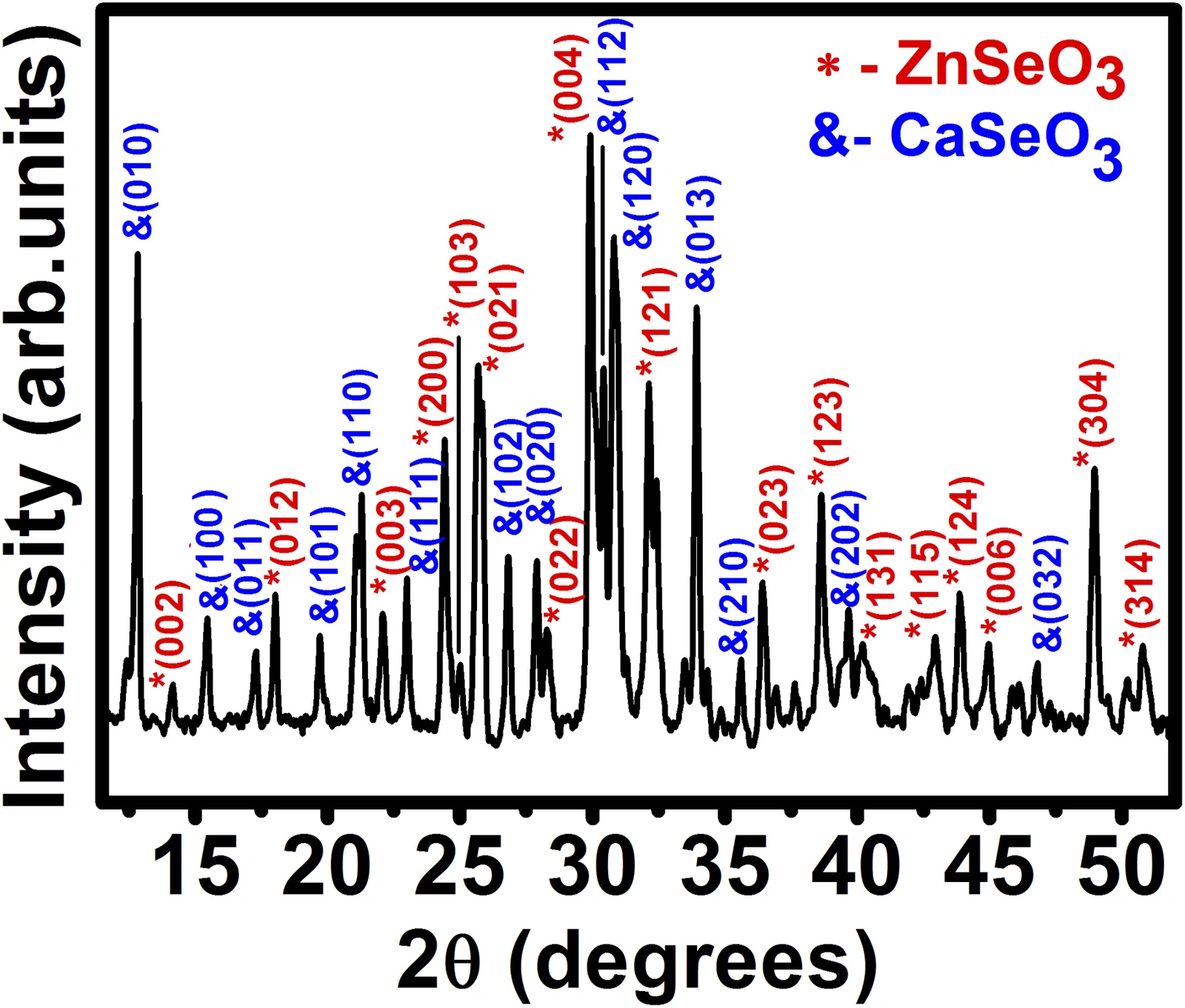
XRD of ZnSeO_3_/CaSeO_3_ nanocomposite.

Fig. 5 depicts the XRD spectra of ZnSeO_3_/CaSeO_3_, which were analysed to determine their structure. The high intensity peaks of ZnSeO_3_ with *hkl* planes of (002), (012), (003), (200), (021), (022), (004), (121), (023), (123), (222), (115), (124), (006), (304), (314) with orthorhombic lattice parameters *a* = 7.188 Å, *b* = 6.225 Å, *c* = 11.976 Å, *α* = *β* = *γ* = 90°. It matches with JCPDS File: 75-0718. ZnSeO_3_ crystallizes in the orthorhombic *Pbca* space group. CaSeO_3_ exhibits high intensity planes with the hkl planes of (010), (100), (011), (101), (110), (111), (102), (020), (112), (120), (013), (210), (122), (032) with orthorhombic lattice parameters *a* = 5.39 Å, *b* = 6.43 Å, *c* = 8.34 Å, *α* = *β* = *γ* = 90°^20^ (JCPDS File: 35-0885; 35-0884). CaSeO_3_ crystallizes in the orthorhombic *Pnma* space group [https://new.ddl.ae/book/9932625]. Sharp peaks confirm crystalline character. Peaks are indexed with the JCPDS file. The crystallite size deduced by the Debye–Scherrer formula is found to be 26.312 nm.

### Optical studies

**Fig. 6:**
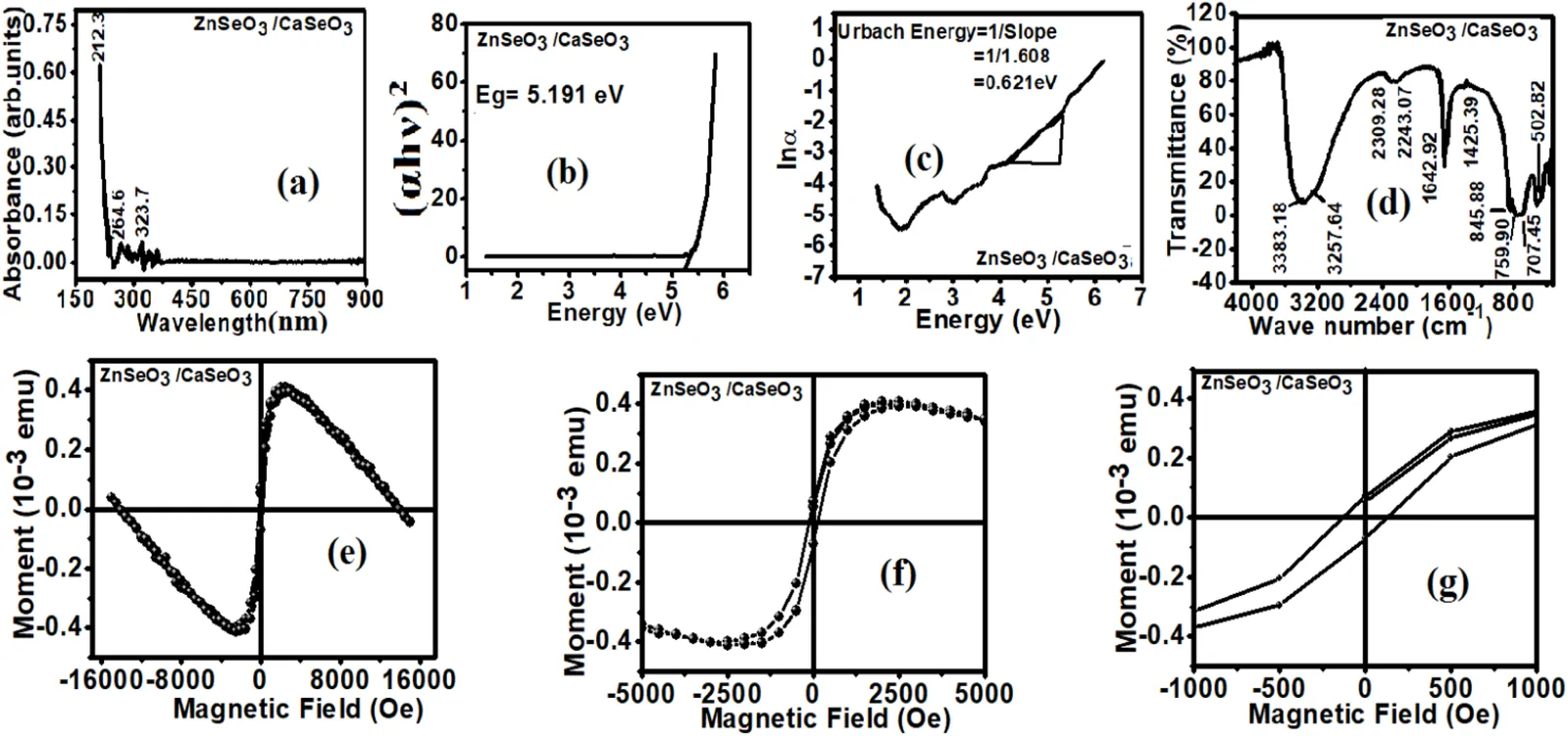
(a) UV absorbance spectrum, (b) Tauc diagram, (c) Urbach graph, (d) FTIR, (e) *M*–*H* curve, and (f) enlarged *M*–*H* curve between ±5000 Oe and (g) Enlarged *M*–*H* curve between ±1000 Oe for the ZnSeO_3_/CaSeO_3_ nanocomposite.

The strong UV absorption at 212.3 nm in the UV-Visible spectrum (Fig. 6a) of ZnSeO_3_/CaSeO_3_ made it a suitable UV filter.^21^ The peak at 264.6 is due to the π–π* transition, and the peak observed at 323.7 nm arises due to the electron transition Zn_3d_ → O_2p_.^22^ The broad transmittance from 250 to 900 nm makes this material a strong optoelectronic candidate.^21^ The decrease in the absorption peak indicates the aggregation of generated nanoparticles, and the peak at 212.3 nm is at the boundary of the UV region, which can be explained by the smaller size of the nanoparticles. It has antioxidant qualities that help prevent UV-induced damage and photocatalytic activity.^23,24^ The optical energy band gap is obtained as 5.191 eV from the Tauc plot (Fig. 6b). Because of various surface and interface defects in nanocomposites, additional sub-band-gap energy levels exist that cause a change in energy between the conduction and valence bands. It is a promising material for display technologies because of native point defects and Schottky and Frenkel surface defects, which promote radiative electron–hole recombination.^25,26^ The calculated Urbach energy from the Urbach plot (Fig. 6c) is 0.621 eV, which shows the characteristic energy associated with the absorption edge in the disordered semiconductor material. It represents the width of localized states extending into the band gap due to structural disorder. Based on the relationship below, the refractive index 1.972 is obtained from the optical energy gap.^27,28^
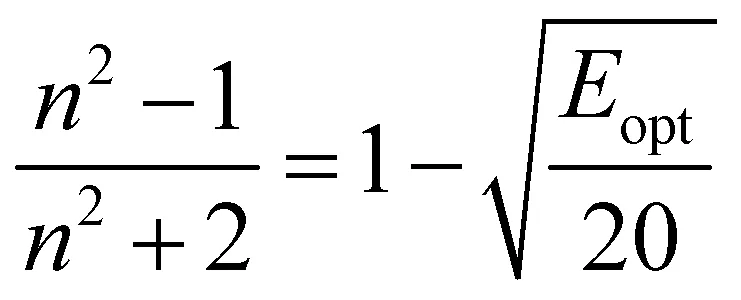


Fig. 6d shows the FTIR spectrum of ZnSeO_3_/CaSeO_3_. It analyses the molecular structures and functional groups of a compound. It provides crystal vibration modes and the position of ions in the crystal. Table 1 presents assignments of bands. The O–H stretching is responsible for a wide trough between 3672.93 cm^−1^ and 2579.26 cm^−1^ that shows two troughs at 3383.18 cm^−1^ and 3257.64 cm^−1^.^29,30^ The O–H bond is responsible for the peak at 2309.28 cm^−1^, while the potential bonding of Zn in ZnO is responsible for the peak at 2243.07 cm^−1^.^31^ At 1642.92 cm^−1^ and 1425.9 cm^−1^, the peak is ascribed to the O–H bond's bending vibration, indicating the sample's hygroscopic nature.^32^ The Se–O bond is shown by the peak at 845.88 cm^−1^.^33^ ZnO is confirmed to be present in the sample by peaks at 759.90 and 707.45 cm^−1^.^34^ Zn–Se vibrations are responsible for the distinctive peak that appears at 502.82 cm^−1^.^35^

**Table 1:** FTIR assignments of bands

S. no	Wavenumber (cm^−1^)	Band assignments
1	3383.18	O–H vibration
2	3257.64	O–H vibration
3	2309.28	O–H vibration
4	2243.07	Zn–O vibration bond
5	1642.92	O–H bending vibration
6	1425.39	O–H bending vibration
7	845.88	Se–O vibration bond
8	759.90	Zn–O vibration bond
9	707.45	Zn–O vibration bond
10	502.82	Zn–Se vibration bond

### Magnetic studies

The magnetization of ZnSeO_3_/CaSeO_3_ is determined with various applied fields *H* up to ±15 000 Oe. Fig. 6e illustrates the fluctuation of magnetization with the applied field at ambient temperature using a vibrating magnetometer.^36^ Interestingly, the hysteresis curve is smooth in the first and third quadrants. Ferromagnetic properties dominate at lower magnetic fields ≤ ±5000 Oe (Fig. 6f). The combination of missing bonds and distortion of the lattice in nanoparticles is responsible for ferromagnetic behaviour in the lower field region. The expanded plot, Fig. 6g, ranging between ±1000 Oe, displays the magnetization behavior of ZnSeO_3_/CaSeO_3_, which seems to be weakly ferromagnetic. Furthermore, it is suggested that a decrease in particle size may be the cause of the weak ferromagnetism.^36,37^

### Thermal analysis

**Fig. 7:**
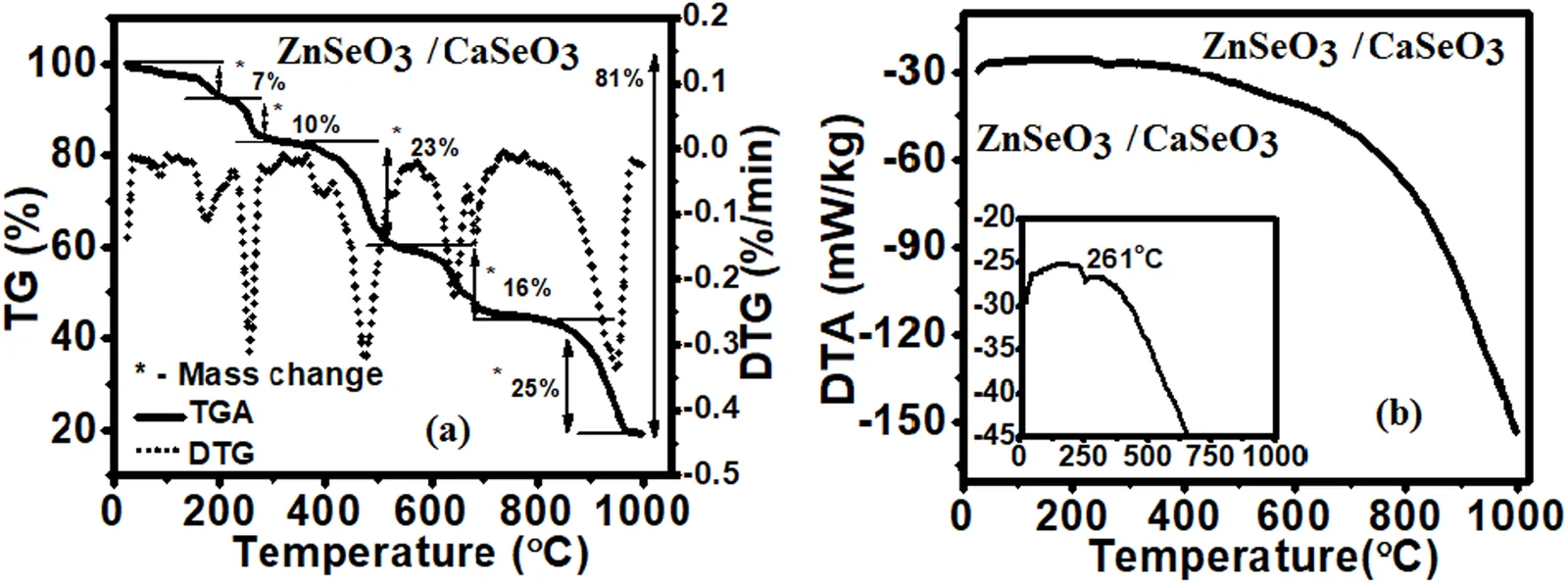
(a) TGA and DTG plots and (b) DTA curve of ZnSeO_3_/CaSeO_3_ nanocomposite.

TGA curve of ZnSeO_3_/CaSeO_3_ is depicted in Fig. 7a. Thermogravimetric analysis (TGA) measures mass changes of material with temperature. The *x*-axis displays temperature from 26 °C to 1000 °C. TG (%), which ranges from 20% to 100% and represents the amount of mass that remains as temperature increases, is displayed on the left *y*-axis.^38^ The mass change is found to be 7% between 26 °C and 211 °C, 10% between 211 °C and 321 °C, 23% between 321 °C and 531 °C, 16% between 531 °C and 811 °C, and 25% between 811 °C and 991 °C. The overall mass change of 81% in TGA indicates that the initial mass of the sample has been lost as a function of temperature, and it is generally due to the release of volatile components, decomposition, oxidation, absorption, or desorption of the material. Non-volatile residues, like stable inorganic components or ash, make up the remaining 19% at the final temperature.^39^ Weight loss per minute is plotted against temperature in the DTG curve (right *y* axis). The graph's peaks correspond to weight loss when the rate of mass loss changes significantly. Due to specific temperature parameters, including breakdown and phase transitions, these peaks show considerable weight loss.^40^ While the DTG peaks show critical temperatures that result in significant loss of weight, the TGA curve shows the sample's thermal stability and breakdown behavior. The general pattern points to multiple phases of volatilization or disintegration.^41^

The DTA curve of ZnSeO_3_/CaSeO_3_ is shown in Fig. 7b with an enlarged plot as an inset. DTA is a technique that measures the change in temperature between the sample and reference substance as a function of the applied temperature. The *x*-axis indicates the temperature (°C) of the furnace from 0 °C to 1000 °C in order to analyse the behavior of the material against temperature. The *y*-axis indicates the difference in temperature between the sample and reference material in milliwatts per milligram (mW mg^−1^).^42^ At lower temperatures up to 250 °C, the curve appears flat with slight and unnoticeable shifts. This suggests no thermal changes occurred during this range.^43^ The peak at 261 °C corresponds to the decomposition of the material and reveals the endothermic nature of the thermal decomposition process. From 300 °C to 600 °C, the graph is flat, which does not show any exothermic or endothermic nature between these regions. Beyond 600 °C, the graph gradually slopes downwards, suggesting an endothermic process in which heat is absorbed by the substance, resulting in melting or decomposition.^44^

### X-ray photoelectron spectroscopy

**Fig. 8:**
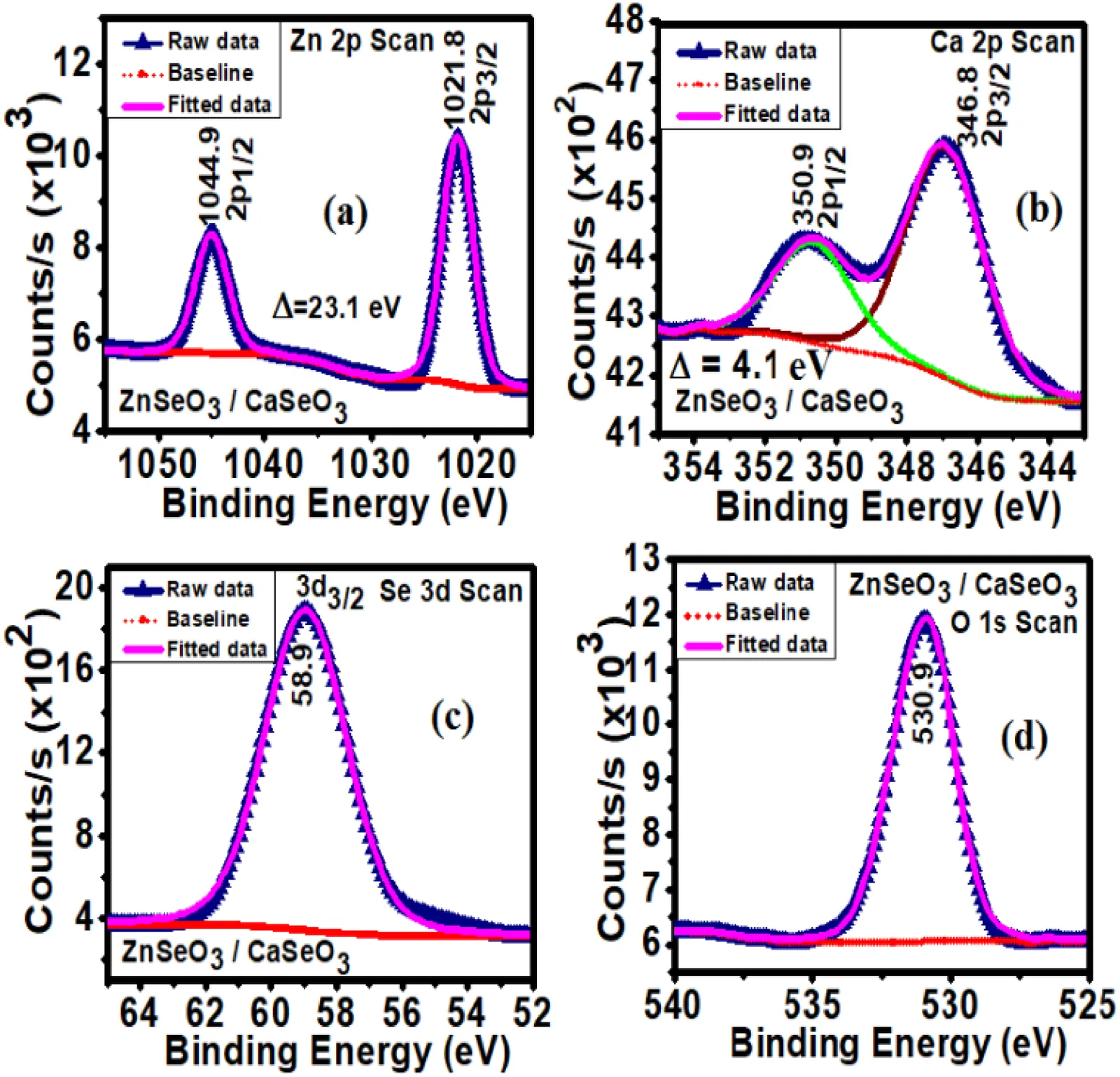
(a) Zinc 2p scan, (b) selenium 3d scan, (c) tellurium 3d scan, (d) oxygen 1s scan of ZnSeO_3_/CaSeO_3_ nanocomposite.

XPS is used for analyzing the surface chemical compositions of materials. Hence, XPS spectra were recorded to obtain more structured information from ZnSeO_3_/CaSeO_3_ experimental results.^45^ XPS spectra of Zn 2p show a doublet for Zn 2p_3/2_ and 2p_1/2_ core levels in Fig. 8a at approximately 1021.8 eV and 1044.9 eV (vertical reference lines).^46^ Zn^2+^ ions oxygen-deficient matrix of ZnO are responsible for the first peak.^47^ The strong Zn 2p_3/2_ XPS peak indicates that the Zn element is mostly present on the sample surface as Zn^2+^. That means ZnO actually forms the samples' capped region.^48^ Spin–orbit splitting with the binding energy of about 23.1 eV shows the p tetrahedral Zn^2+^ existence, confirming that Zn oxidation state is +2 in ZnSeO_3_/CaSeO_3_.^49^ Ca (2p) spectrum represents Ca 2p_3/2_ and 2p_1/2_ binding energies at 346.8 eV and 350.9 eV, as in Fig. 8b. However, the chemical shift of Ca 2p_3/2_ and 2p_1/2_ differs by 4.1 eV. This energy difference likely corresponds to charge transfer or interband transition in the electronic structure.^50^ Upon contact with air, the oxidation of the metal selenite surface produces a significant peak centered at 58.9 eV in the Se 3d_3/2_ scan (Fig. 8c), aligning with existing literature for Se in the selenite (SeO_3_) form. This summit is assigned to the Se–O bond.

**Fig. 9:**
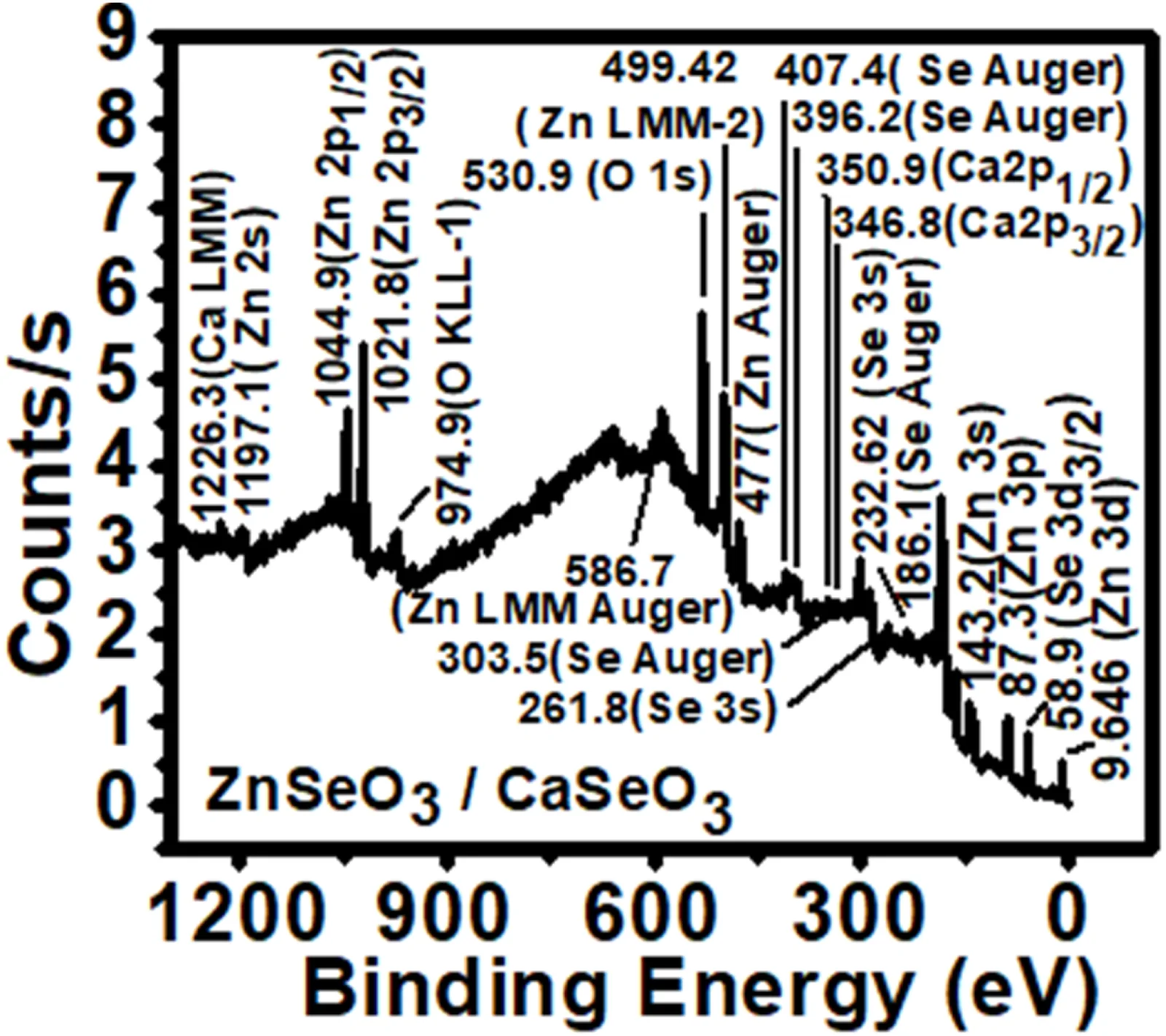
XPS survey spectrum of ZnSeO_3_/CaSeO_3_.

This could be due to surface oxidation or indicate that selenium is in a higher oxidation state.^51^ The shape and position of the peak indicate chemical characteristics of selenium in the sample.^52^ The peak position of low energy at 530.9 eV in the O 1s scan (Fig. 8d) is unaltered.^53^ This peak represents the deepest electron shell of oxygen atoms, or the O 1s core level. The peak position indicates the substance's oxygen chemical environment. Different oxygen 1 s peaks are seen in different chemical states, such as oxides, hydroxides, or absorbed species. O^2−^ in the ZnO hexagonal wurtzite structure has been linked to a peak with a lower binding energy of roughly 530.9 eV, which is attributed to a larger concentration of oxygen vacancies linked to lattice instability.^54,55^ The survey spectrum indicates that the element's atoms' chemical and electrical states are visible on the surface between 1 and 10 nm. The electron counts and binding energy (eV) are plotted along the *y*-axis and the *x*-axis, respectively. The chemical element in the top 10 nm of the surface can be identified using this method.^56^ The ZnSeO_3_/CaSeO_3_ survey spectrum is illustrated in Fig. 9, confirming the presence of zinc, calcium, selenium, and oxygen.^57^

### Electrochemical analysis of ZnSeO_3_/CaSeO_3_ in a three-electrode setup

**Fig. 10:**
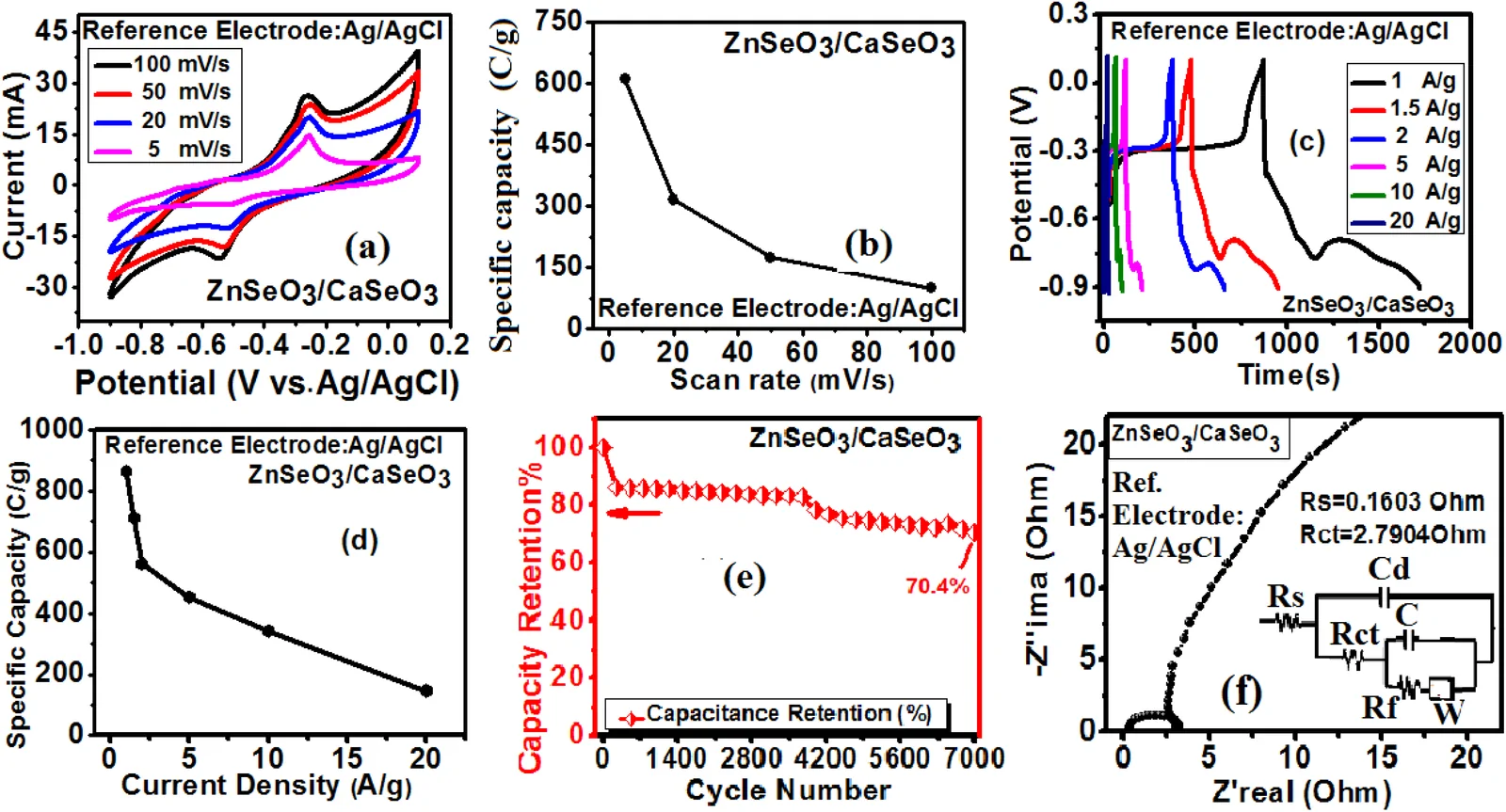
(a) CV graph, (b) specific capacity *versus* scan rate, (c) charge–discharge curves, (d) specific capacity as a function of current density, (e) cyclic stability, and (f) Nyquist plot of ZnSeO_3_/CaSeO_3_ nanocomposite.

Supercapacitive properties of ZnSeO_3_/CaSeO_3_ electrodes are studied to determine their applicability in the supercapacitor field. 3 M KOH electrolyte is used in electrochemical analysis by the standard three-electrode setup. Cyclic voltammetric (CV) responses have been recorded in a 1 V potential range, 0.9 to 0.1 V. The CV of ZnSeO_3_/CaSeO_3_ in Fig. 10a shows two redox peaks at −0.25 V and −0.5 V, suggesting that the charge storage mechanism was caused by quick faradaic redox processes. Interestingly, the CV profile exhibits a shift in peaks, which is explained by internal resistance generated in the electrode material and insufficient electrolyte ion neutralization. CV curves exhibit distinct voltage plateaus during charging and discharging instead of the perfectly linear, triangular voltage–time slope seen in typical capacitor or pseudocapacitor materials, which confirms that the material is a battery-type material. Moreover, CV displays well-defined redox peaks, which correspond to the oxidation and reduction of active material, as in battery-type material. To definitively differentiate a battery-type diffusion-controlled process from surface-controlled capacitive behaviour, one must analyse the relation between scan rate (*v*) and peak current (*i*_p_):*i*_p_ = *av*^*b*^

The *b*-value analysis determines *b* = 0.5. It confirms battery-type diffusion-controlled kinetics, which means that the rate of charge storage is limited by diffusion of ions into the bulk^58,59^

At sweep rates of 5, 20, 50, and 100 mV s^−1^, the ZnSeO_3_/CaSeO_3_ in the electrode displays outstanding specific capacities of 610.14, 315.88, 173.74, and 99.46C g^−1^, respectively. Specific capacity *Q*s of electrode material decreases with rising scan rate, as seen in Fig. 10b. The CV graph deduces specific capacitance *C*_s_ from the following equation:
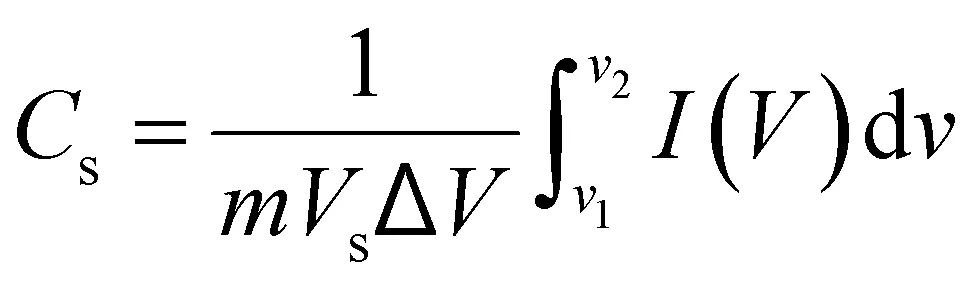
Specific capacity *Q*_s_ = *C*_S_ × Δ*V*where the values *m*, *I*(*v*) d*v*, *v*_1_, *v*_2_, Δ*V*, and *V*_s_, respectively, denote active material mass (g), area of current density (A cm^−2^), lower and higher potential cut off, potential window (V), and scan rate (mV s^−1^).^60^ These results suggest that the composite electrode has superior electrochemical activity and capacity. This occurs because at lower scan rates, ions from the electrode/electrolyte interface interact with both the outer and inner surfaces of the electrode, but at high scan rates, ions lack sufficient time to reach both surfaces. Furthermore, prepared electrodes' charge capability, rate performance, and durability were evaluated using the galvanostatic charge/discharge method. Charge–discharge characteristics were measured for various current densities (1–20 A g^−1^) in a potential range of 1 V (−0.9 V to 0.1 V). The GCD plateaus of ZnSeO_3_/CaSeO_3_ tested at various densities of current are shown (Fig. 10c), indicating that the redox reaction was primarily responsible for the capacity of the electrode material, which is a non-linear plot. The slow-kinetic process over the active material is confirmed by the non-linear charge–discharge curve.^61^ In addition, the electrode material's specific capacity was evaluated by analyzing the discharge area of the GCD profile, using
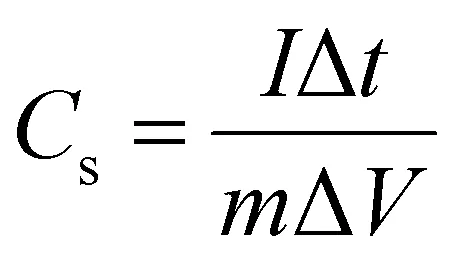
Specific capacity *Q*_s_ = *C*_S_ × Δ*V*where Δ*t*, *I*, Δ*V*, and *m* stand for discharge duration (s), discharge current (A), potential difference (V) during discharge, and active material mass (g), respectively.^60^ The computed maximum specific capacity was 862.81 F g^−1^ at 1 A g^−1^ low current density. The existence of a porous structure, which provides continuous pathways for the flow of electrolyte ions, is the cause of this remarkable capacitive feature. Specific capacity deduced from GCD (Fig. 10d) is 711.49, 559.74, 452.73, 341.52, and 146.80 F g^−1^ at 1.5, 2, 5, 10, and 20 A g^−1^ current densities. It shows that current density rises, with a fall of electrode materials' capacity (Fig. 10d). The two primary causes are IR drop at high current density and internal resistance of the electrode material. Excellent efficiency is promoted by ions having enough time to intercalate at a lower current. Unfortunately, inadequate time at high current densities has limited the migration and intercalation of electrolyte ions, leading to poor electrochemical performance. An abnormal inflection point is observed in Fig. 10(c), which may be explained as follows. The GCD displays a nonlinear profile with pronounced voltage plateaus or distinct inflections or voltage shoulders during the charge/discharge process, indicating that charge storage is governed by battery-type faradaic reactions. The presence of plateau regions or inflection points indicates reversible oxidation–reduction reactions occurring at specific potentials, and they are responsible for high charge-storage capacity. During the charging process, the potential increases gradually before reaching a distinct plateau, suggesting the occurrence of electrochemical oxidation of the active material. Similarly, during discharge, the corresponding plateau reflects the reverse reduction reaction. These plateaus represent phase or oxidation-state transformations associated with ion insertion/extraction from the electrode. The initial voltage drop at the beginning of the discharge is indicative of an ohmic (IR) drop. It arises from the internal resistance of the electrode/electrolyte system. Moreover, if the material contains mixed-valence elements or two electrochemically active phases such as ZnSeO_3_/CaSeO_3_, sequential redox reactions can produce shoulders or inflection points. Battery-type materials often undergo phase transitions during ion insertion/extraction, producing an intermediate plateau or inflection.^62–64^

Moreover, at 5 A g^−1^ current density, 7000 charge–discharge cycles were conducted to evaluate the cyclic lifespan of electrodes (Fig. 10e) to assess stability. A small fluctuation in specific capacity (the activation of electrode material) has been seen for the first 2000 cycles. Specific capacity then slowly drops during the course of the following 2000 cycles. There was a decline in specific capacity after 5000 cycles. The electrode material shows 70.4% retention after 7000 cycles continuously.^65^

The electrochemical impedance spectroscopy was used to measure the ionic, resistive, and capacitive properties of the ZnSeO_3_/CaSeO_3_ electrodes. The impedance spectrum is split into two sections: (i) the low-frequency portion (linear region) is linked to Warburg resistance, and (ii) the high-frequency area (semi-circle region) indicates charge transfer resistance caused by the Faradaic redox process. Based on the electrochemical impedance spectrum (Fig. 10f), charge transfer resistance is 2.7904 Ω, and equivalent series resistance is 0.1603 Ω using the modified Randle's circuit. This implies that the electrochemical performance of the generated nanocomposite is probably enhanced by a reduced electrode electrolyte solution resistance, which is indicated by a lower charge transfer resistance. The impedance spectrum shows a narrow semicircle, which indicates strong electrical conductivity between the working electrode and the current-collecting electrode and charge-transfer resistance *R*_ct_ = 2.7904 Ω. The large rise in capacity explains this. Warburg impedance (Fig. 10f) is shown as a straight line with a 45° slope under semi-infinite conditions. In Fig. 10f, the comparable circuit is displayed as an inset. The Warburg element, ZWAR, contains the species diffusion coefficient. Higher mass transfer and electrolyte diffusion rates are suggested by an ideal straight line in the low-frequency area at around a 45° slope from the real axis, because of its excellent ionic diffusion and electrolyte access, great electrical conductivity, and extremely low internal resistance. This suggests that low charge transfer resistance likely improves the produced nanocomposite's electrochemical performance. This low *R*_ct_ value greatly improves the electrode material's conductivity and electrochemical performance.

## Conclusions

A metal oxychalcogenide ZnSeO_3_/CaSeO_3_ nanocomposite is synthesized by the hydrothermal technique, and its formation is confirmed. It shows a capsule-like nanorod morphology, enhancing surface area and contributing to improved electrochemical performance. Particle size was 109.4 nm. The composite ZnSeO_3_/CaSeO_3_ is found to be orthorhombic in structure. Its energy gap, Urbach energy, and refractive index are 5.191 eV, 0.621 eV, and 1.972, respectively. The Zn–O and Zn–Se bands are assigned. At 300 K, the sample exhibits modest ferromagnetism. The final breakdown of the remaining (OH^−^) groups and heat release as the oxide lattice forms are shown by endothermic and exothermic reactions seen in the DTA curve. In line with the reference material, it displays the sample's response to temperature in a furnace. Although the TGA curve displays the sample's thermal stability and breakdown behavior, DTG peaks show several stages of disintegration or decomposition. XPS confirms the nanocomposite's chemical condition. The chemical state of the elements in the composite is confirmed by the survey spectrum's peak values. Zn, Se, Te, and O binding energy values ensure an element's orbital state. The three-electrode CV curves show an operating potential window of 1 V. The ZnSeO_3_/CaSeO_3_ cyclic voltammogram shows two redox peaks, indicating that the charge storage process is accomplished by the rapid faradaic redox phenomena. The ZnSeO_3_/CaSeO_3_ electrode exhibits excellent specific capacity of 862.81C g^−1^ at a 1 A g^−1^ current density, which implies that the capacity and electrochemical activity of the composite electrode are superior. Electrode material exhibits 70.4% retention after continuous 7000 charge–discharge cycles at a 5 A g^−1^ current density. Pseudocapacitor behavior is confirmed by the Nyquist plot. It is discovered that the series resistance *R*_s_ is 0.2548 Ω and charge transfer resistance *R*_ct_ is 2.7904 Ω, both of which are low, which enhances electrochemical performance.

## Ethical statement

This is an observational study. No ethical approval is required.

## Author contributions

S. Ariponnammal: conceptualization, analysis, investigation, methodology, writing, editing, guiding, supervision, R. Harshinee: conceptualization, data analysis, investigation, writing draft, review and analysis, S. Shanmugha Soundare: writing, editing, data collection, analysing electrochemical performance data, resources, draft preparation, M. Mithren: writing – editing, analysis, investigation. All authors participated in the manuscript preparation. All authors read and approved the final manuscript.

## Conflicts of interest

The authors declare no conflicts of interest.

## Data Availability

All data generated or analyzed during this study are included in this article.

## References

[cit1] Sahin M. E., Blaabjerg F., Sangwongwanich A. (2022). Energies.

[cit2] Ang T. Z., Salem M., Kamarol M., Das H. S., Nazari M. A., Prabaharan N. (2022). Energy Strategy Rev..

[cit3] Kardooni R., Yusoff S. B., Kari F. B. (2016). Energy policy.

[cit4] Manohar A., Vijayakanth V., Vattikuti S. V. P., Kim K. H. (2023). Ceram. Int..

[cit5] Sharma K., Arora A., Tripathi S. K. (2019). J. Energy Storage.

[cit6] Wang S., Wei F., Geng W., Ren Z., Lv Y. (2025). Chem. Eng. J..

[cit7] Wei F., Zhang H., Wang J., Zhuang J., Lv Y. (2022). J. Alloys Compd..

[cit8] Sun Y., Zhang G., Tan S., Wang H., Liu Y., Sun C., Deng B., Zhang K., Gao Z., Huang N. (2025). Chem. Eng. J..

[cit9] Sun C., Fang Z., Wang H., Ge M., Zhang S., Zhang K., Deng B., Wang J., Sun Y. (2026). J. Alloys Compd..

[cit10] Sun Y., Wang H., Ge M., Tan S., Deng B., Zhang K., Zhang H., Zhan S., Sun C., Wang X. (2026). Chem. Eng. J..

[cit11] Tan S., Liu Y., Sun X., Zhang S., Sun C., Sun Y., Huang N. (2025). New J. Chem..

[cit12] Sun Y., Ge M., Xie B., Zhang H., Gao Z., Zhao X., Deng B., Zhang K., Zhang S., Sun C., Wang X. (2025). J. Energy Storage.

[cit13] Ge M., Sheng Y., Fang Z., Jiang W., Yan J., Lu F., Sun C., Sun Y. (2025). CrystEngComm.

[cit14] Raza W., Ali F., Raza N., Luo Y., Kim K. H., Yang J., Kumar S., Mehmood A., Kwon E. E. (2018). Nano Energy..

[cit15] Sahin M. E., Blaabjerg F., Sangwongwanich A. (2020). Turk. J. Chem..

[cit16] Lacroix J. C., Kanazawa K. K., Diaz A. (1989). J. Electrochem. Soc..

[cit17] Archana J., Navaneethan M., Ponnusamy S., Hayakawa Y., Muthamizhchelvan C. (2009). Mater. Lett..

[cit18] Anitha A., Ponmurugan P., Arunkumar D., Sumathi C. S., Sathishkumar M., Purushothaman T. (2025). BioMetals.

[cit19] Agrawal A., Siddiqui S. A., Soni A., Sharma G. D. (2025). Discover Appl. Sci..

[cit20] https://next-gen.materialsproject.org/materials/mp-754140#CTMaterialsApp_crystal_structure

[cit21] Tahenti M., Issaoui N., Roisnel T., Kazachenko A. S., Iramain M. A., Brandan S. A., Al-Dossary O., Kazachenko A. S., Marouani H. (2023). Z. Phys.Chem..

[cit22] Verma P. R., Khan F., Banerjee S. (2020). Inorg. Nano-Met. Chem..

[cit23] Liu Q., Zhu C., Xie G., Wang J., Zhang D., Shao G. (2022). J. Inorg. Mater..

[cit24] Shar A. H., Lakhan M. N., Wang J., Ahmed M., Alali K. T., Ahmed R., Ali I., Dayo A. Q. (2019). Dig. J. Nanomater. Biostruct..

[cit25] Kislov N., Srinivasan S. S., Emirov Y., Stefanakos E. K. (2008). Mater. Sci. Eng., B.

[cit26] Kumar K. V. (2022). Adv. Mater. Phys. Chem..

[cit27] Halimah M. K., Faznny M. F., Azlan M. N., Sidek H. A. A. (2017). Results Phys..

[cit28] Saudi H. A., Adel G. (2018). Optics..

[cit29] Jansook P., Ritthidej G. C., Ueda H., Stefánsson E., Loftsson T. (2010). J. Pharm. Pharm. Sci..

[cit30] Elrefaey A. A. K., El-Gamal A. D., Hamed S. M., El-Belely E. F. (2022). Egypt. J. Chem..

[cit31] Jeronsia J. E., Joseph L. A., Jaculine M. M., Vinosha P. A., Das S. J., Taibah J. (2016). Univ. Sci..

[cit32] Rahman S., Maria K. H., Ishtiaque M. S., Nahar A., Das H., Hoque S. (2020). Turk. J. Chem..

[cit33] Ralph N. G., Soundare S. S., Harshinee R., Ariponnammal S. (2024). Chalcogenide Lett..

[cit34] Bashir S., Awan M. S., Farrukh M. A., Naidu R., Khan S. A., Rafique N., Ali S., Hayat I., Hussain I., Khan M. Z. (2022). Int. J. Nanomed..

[cit35] Ahamed A. J., Ramar K., Kumar P. V. (2016). J. Nanosci. Technol..

[cit36] Roy R., Singh D., Kumar M. S. (2023). J. Mater. Sci.: Mater. Electron..

[cit37] Elsi S., Pushpanathan K. (2019). J. Mater. Sci.: Mater. Electron..

[cit38] Emiola-Sadiq T., Zhang L., Dalai A. K. (2021). ACS omega.

[cit39] Van Riessen A., Rickard W., Sanjayan J. (2009). Geopolymers. Chapter.

[cit40] Aparicio-Rojas G. M., Medina-Vargas G., Díaz-Puentes E. (2020). Heliyon.

[cit41] https://resources.perkinelmer.com/lab-solutions/resources/docs/FAQ_Beginners-Guide-to-Thermogravimetric-Analysis_009380C_01.pdf

[cit42] Kalai F. El., Çınar E. B., Lai C. H., Daoui S., Chelfi T., Allali M., Dege N., Karrouchi K., Benchat N. (2021). J. Mol. Struct..

[cit43] Xiong G., Pal U., Serrano J. G., Ucer K. B., Williams R. T. (2006). Phys. Status Solidi C.

[cit44] De la Fuente J. L., Ruiz-Bermejo M., Menor-Salván C., Osuna-Esteban S. (2011). Polym. Degrad. Stab..

[cit45] Wang F., Liu J., Wang Z., Lin A. J., Luo H., Yu X. (2010). J. Electrochem. Soc..

[cit46] Lupan O., Emelchenko G. A., Ursaki V. V., Chai G., Redkin A. N., Gruzintsev A. N., Tiginyanu I. M., Chow L., Ono L. K., Cuenya B. R., Heinrich H. (2010). Mater. Res. Bull..

[cit47] Huang S. Y., Cheng Q. J., Xu S., Wei D. Y., Zhou H. P., Long J. D., Levchenko I., Ostrikov K. (2012). J. Appl. Phys..

[cit48] Morozov I. G., Belousova O. V., Ortega D., Mafina M. K., Kuznetcov M. V. (2015). J. Alloys Compd..

[cit49] Li Z., Chen H., Liu W. (2018). Catalysts.

[cit50] Chu D. H., Vinoba M., Bhagiyalakshmi M., Baek I. H., Nam S. C., Yoon Y., Kim S. H., Jeong S. K. (2013). RSC Adv..

[cit51] Huang J., Li X., Jiang S., Liu C., Lu M., Yang Y. (2022). J. Electron. Mater..

[cit52] Chubar N. (2023). J. Environ. Chem. Eng..

[cit53] Zhang H. L., Wang D. Z., Huang N. K. (1999). Appl. Surf. Sci..

[cit54] Gordillo G., Pena J. C. (2022). J. Mater. Res. Technol..

[cit55] Mahmoud A. Z., Ibrahim E. M. M., Galal L., Shaaban E. R., Yousef E. S., Ovonic J. (2023). Res..

[cit56] Gayathri R., Soundare S. S., Mithren M., Ariponnammal S., Ovonic J. (2025). Res..

[cit57] Chastain J. and King Jr R. C., Handook of x-ray photoelectron spectroscopy, Perkin-Elmer Corporation, 1992

[cit58] Guo B., Gao Y., Li Y., Liu K., Lv X., Mi C., Liu L., Li M. (2023). ACS Omega.

[cit59] Aldama I., Barranco V., Ibã nez J., Amarilla J., Rojo J. M. (2018). J. Electrochem. Soc..

[cit60] Sethi M., Sandhya Shenoy U., Bhat D. K. (2020). Nanoscale Adv..

[cit61] Dhanalakshmi S., Vathani A. M., Muthuraj V., Prithivikumaran N., Karuthapandian S. (2020). J. Mater. Sci.: Mater. Electron..

[cit62] Conway B. E. , Electrochemical Supercapacitors: Scientific Fundamentals and Technological Applications, Kluwer Academic/Plenum Publishers, 1999

[cit63] Guo B., Gao Y., Li Y., Liu K., Lv X., Mi C., Liu L., Li M. (2023). ACS Omega.

[cit64] Aldama I., Barranco V., Ibã nez J., Amarilla J. M., Rojo J. M. (2018). J. Electrochem. Soc..

[cit65] Verma S., Khosla A., Arya S. (2020). J. Electrochem. Soc..

